# ﻿The re-description of *Synoecnemahirsutum* Timm, 1959 (Synoecneminae, Ungellidae, Drilonematoidea) from a pheretimoid earthworm in Vietnam with the analysis of its phylogenetic relationships

**DOI:** 10.3897/zookeys.1076.75932

**Published:** 2021-12-09

**Authors:** Elena S. Ivanova, Boris D. Efeykin, Sergei E. Spiridonov

**Affiliations:** 1 Centre of Parasitology of the Severtsov Institute of Ecology & Evolution, Russian Academy of Sciences, Leninski pr. 33, Moscow 119071, Russia Centre of Parasitology of the Severtsov Institute of Ecology and Evolution, Russian Academy of Sciences Moscow Russia; 2 Joint Russian-Vietnamese Tropical Scientific and Technological Center, Cau Giay, Hanoi, Vietnam Joint Russian-Vietnamese Tropical Scientific and Technological Center Hanoi Vietnam

**Keywords:** Drilonematids, ungellids, earthworms, Vietnam, mitochondrial genome, phylogeny

## Abstract

*Synoecnemahirsutum* Timm, 1959 (Ungellidae, Drilonematoidea), found in the body cavity of the pheretimoid earthworm at the border of Laos and Vietnam, was re-described and illustrated. The mitochondrial genome of *S.hirsutum* obtained with Illumina HiSeq sequencing is the first annotated mitochondrial genome as a representative of the superfamily Drilonematoidea. The phylogeny inferred from the analysis of 12 mitochondrial genes has shown some similarity of *S.hirsutum* with a cephalobid *Acrobeloidesvarius*.

## ﻿Introduction

Recent phylogenetic analyses have shown that multiple (18 according to Viney 2017) separate occasions of transition to parasitic lifestyle had occurred in the evolutionary history of nematodes. Phylogenetic relationships of parasitic nematodes and free-living representatives of this phylum can reveal the evolutionary pathways of the acquisition of parasitism in this group of animals. However, the evolutionary history of the most numerous group of parasitic nematodes – the order Rhabditida (formerly the subclass or the class Secernentea) still remains a puzzle. The analysis of contemporary literature demonstrates that different groups of parasites have been studied in a very inconsistent manner with the bias on the parasitic nematodes of humans, domesticated animals and plants versus the parasitic nematodes of economically-unimportant invertebrates. The latter are also poorly presented in the mitochondrial genome phylogenetic research.

It is evident that evolutionary processes of parasitism acquisition are not anchored to the organisms important for humans and the study of neglected groups of parasites and their hosts can reveal interesting evolutionary patterns. One of such groups is the Drilonematoidea, one of the larger nematode taxa with still unresolved phylogeny. It hosts the highly specialised and diverse group of coelomic parasites of earthworms (Annelida, Clitellata). The impressive diversity of the nematodes, parasitic in earthworms, was discovered and described by R.W. Timm (1959, 1962, 1966, 1967), preceded by earlier reports by Dujardin (1845), Cobb (1928), Chitwood and Lucker (1934) and Baylis (1943). A separate superfamily Drilonematoidea was established to incorporate the species using the earthworms as the definitive and single hosts (Chitwood and Chitwood 1950). The first attempts to infer the phylogeny of the earthworm-parasitic nematodes from the analysis of nucleotide sequences demonstrated the polyphyletic nature of Drilonematoidea (Spiridonov et al. 2005; Ivanova and Spiridonov 2011, 2015, 2016). Based on the SSU phylogeny, the Creagrocercidae family was classified into Plectida i.e. outside Rhabditida, while the rest of the superfamily into the infraorder Cephalobomorpha within clade IV according to Blaxter et al. (1998), either to the suborder Tylenchina, together with Panagrolaimorpha and Tylenchomorpha (De Ley and Blaxter 2004). Today, the mitochondrial genome phylogeny is seen as a valuable tool in the resolution of the relationships between taxa (Zou et al. 2017; Kern et al. 2020; Kim et al. 2020), as well as in the understanding of nematode parasitism (Viney 2017). Viney emphasises the importance of comparing related forms with different lifestyles to identify genes or gene families that changed. The Drilonematoidea hosting exclusively parasitic forms represents the separate case within Cephalobomorpha with its majority of free-living forms inhabiting a variety of land habitats. To date, the only cephalobomorph mt complete genome sequenced is that of *Acrobeloidesvarius* Kim, Kim & Park, 2017 (Kim et al. 2020), a typical free-living nematode.

Within Drilonematoidea, the family Ungellidae (ungellids) is the most speciose taxon with the morphology profoundly changed by the parasitic lifestyle. These nematodes are characterised by the presence of cephalic hooks, expanded glandular structures in the enlarged tail portion of the body, degeneration of a spicular apparatus in males and thick-walled eggshells. They are very rare in earthworms inhabiting temperate regions, but found in the earthworm taxa of tropics and subtropics. Such geographical distribution of ungellids is a serious obstacle to the examination of this group with modern techniques.

The species of *Synoecnema* (Ungellidae) was found by S. E. Spiridonov in the body cavity of a pheretimoid earthworm collected in Vietnam at the border with Laos in April 2019. The genus *Synoecnema* had been established by de Magalhães in 1905 to accommodate a nematode species (*S.fragile*) found in the body cavity of an earthworm and characterised by the presence of a pair of cephalic hooks. Its taxonomic status was revised by Baylis and Daubney (1926) who assigned four species of *Dionyx* also recovered from the earthworm coelomic cavities and described by Pierantoni (1916), to *Synoecnema*. Later, Baylis (1943) had considered their decision unjustified because males of at least two of *Dionyx* species featured a spicular apparatus lacked by *S.fragile*. Since *Dionyx* had been a preoccupied name used for a coleopteran, Baylis (1943) proposed the new genus *Onychonema* for the accommodation of Pierantoni’s *D.cognetti*, *D.minuta*, *D.guinensis* and *D.acutifrons*, leaving *S.fragile* as the type species of *Synoecnema* and describing four more species of the latter. Further on, Timm (1962), revising *Synoecnema*, transferred species *O.acutifrons* and *O.guinensis* back to *Synoecnema* noting that Baylis overlooked the lack of a spicular apparatus in the *O.acutifrons* male attached to a female in a permanent copula and the great similarity of *O.guinensis* females to that of all known species of *Synoecnema*. *Onychonema* Baylis, 1943 with its remaining *O.cognetti* and *O.minutum* was considered *insertae sedis* by Spiridonov and Ivanova (2005). The latter authors also synonymised *Siconemella* Timm, 1967, erected to accommodate related forms with larger cephalic hooks, with *Synoecnema*.

So far, all species of *Synoecnema* were found in coelomic cavities of tropical earthworms belonging to Megascolecoidae (mainly) and Drawidae and collected in South America, Papua New Guinea, India and Southeast Asia. To date, the genus *Synoecnema* Magalhães, 1905 accounts for 20 species: *S.fragile* Magalhães, 1905, *S.acutifrons* Pierantoni, 1916, *S.anseriforme* Timm, 1959, *S.* (= *Siconemella*) *burmensis* Timm, 1967, *S.drawidae* Baylis, 1943, *S.gatesi* Timm, 1962, *S.guinensis* Pierantoni, 1916, *S.hirsutum* Timm, 1959, *S.hoplochaetellae* Baylis, 1943, *S.laotense* Spiridonov, 1993, *S.modigliani* Ivanova & Spiridonov, 1989, *S.perionychis* Baylis, 1943, *S.pheretimae* Baylis, 1943, *S.* (= *Siconemella*) *philippinensis* Timm, 1967, *S.pingi* Ivanova & Spiridonov, 1989, *S.robustum* Ivanova & Spiridonov, 1989, *S.rodericensis* Ivanova & Spiridonov, 1989, *S.tsiliensis* Ivanova & Spiridonov, 1989, *S.tuliemense* Ivanova and Pham Van Luc, 1989 and *S.watinagii* Ivanova, Sumaya & Spiridonov, 2015.

To date, the latter species is the only member of the genus molecularly characterised. It is suggested that new discoveries in the morphology and genetics of the members of the genus may bring the need for further revision. The quantity of material collected allowed us to obtain enough material to provide the extended molecular analysis aimed at the resolution of relationships between higher taxa of Drilonematoidea, parasites of earthworms.

## ﻿Materials and methods

Nematode material. Earthworms were collected at Muóng Lát, Thanh Hóa Province, VietNam (20°31'N, 104°56'E) in April 2019 and dissected alive. Fourteen specimens of the earthworm host species were examined and all were found infected by 2–80 nematodes (av. 14). All nematodes were presented by adult stages with the majority found in a state of a permanent copula, characteristic to the genus. Nematodes were located in the body cavity along the whole host body with the majority occupying anterior segments, were not attached to the septae, but were nearly immobile and responded to the dissection of the host by slight twitching soon followed by its distortion, bursting and death. For the morphological examination, about 20 specimens were fixed by hot 4% formalin and the rest by 96% ethanol for molecular studies.

Nematodes, preserved in formalin, were processed by anhydrous glycerine for light microscopy as described by Seinhorst (1959). Light microscopic studies and drawings were done using a Nikon Eclipse 200 microscope equipped with a drawing attachment. Illustrations were prepared using a WACOM Intuos A4 USB drawing tablet and Adobe Illustrator CS5. For the SEM studies, formalin-preserved material was dehydrated, critical point-dried and coated with gold. Images were taken on a Tescan CamScan MV 2300 and Mira3 Tescan. The voucher specimen (the female), under accession number 14283, was deposited in the Museum of the Helminthological Collections of the Centre of Parasitology at the Severtsov Institute of Ecology and Evolution, Moscow.

### ﻿Molecular characterisation and phylogenetic analysis

DNA from the frozen nematode samples was isolated using the QiAmp Micro Kit (Qiagen) according to a standard protocol. DNA library preparation was implemented using the NEBNext Ultra II DNA Library Prep Kit for Illumina (New England Biolabs, Ipswich, MA, USA). The DNA quality was checked with Qubit 3.0, final library length distribution and checking for the absence of adapters was performed using Bioanalyzer 2100 (Agilent, Santa Clara, CA, USA). Sequencing was performed on Illumina HiSeq 4000 system with a 150 bp read length at the Skoltech Genomics Core Facility (https://www.skoltech.ru/research/en/shared-resources/gcf-2/).

The quality of raw reads was evaluated using FastQC (Andrews 2010). After filtering by quality, the remaining readings were cleared by removing cDNA synthesis adapters and sequence adapters by processing with the Trimmomatic programme. *De novo* assembly was implemented using Velvet (Zerbino et al. 2010) with the default settings. The resulting contigs were filtered by length and contigs with the most similarity in size to mitochondrial DNA were selected. Assembled sequences of protein-coding genes were checked for internal stops in PCGs manually. The contigs were annotated using the MITOS web server (Bernt et al. 2013), with the default settings. Prediction of protein-coding genes and rRNA genes was done by using a combination of BLAST and MITOS online software. Concatenated nucleotide alignment of 12 protein-coding genes was performed using GENEIOUS PRIME 2019.1 (Biomatters Ltd., Auckland, New Zealand).

**Figure 1.**
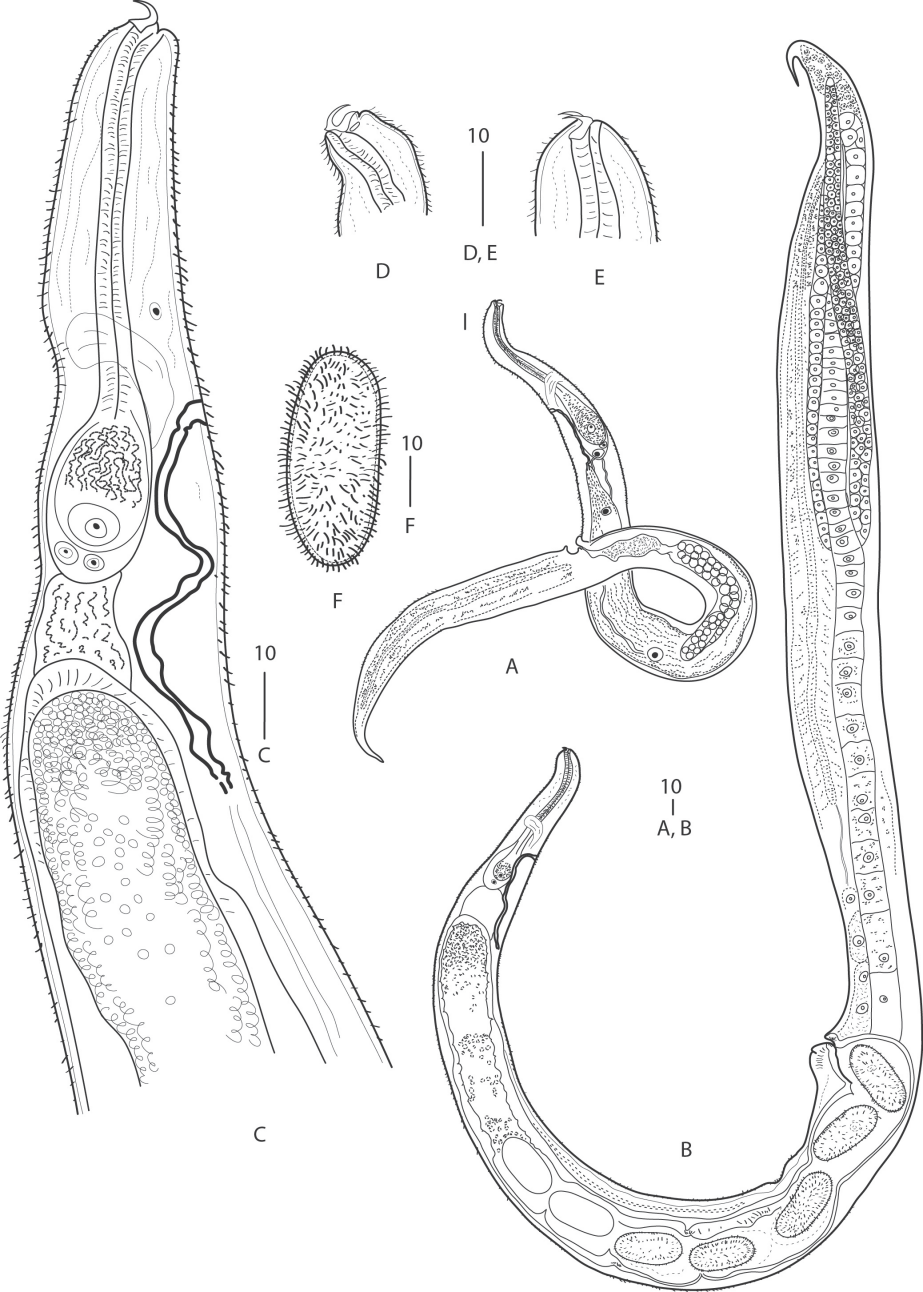
*Synoecnemahirsutum* Timm, 1959 **A** entire male **B** entire female **C** pharynx region of female **D-E** head region of females **F** egg. All in lateral position. Scales in µm.

**Figure 2.**
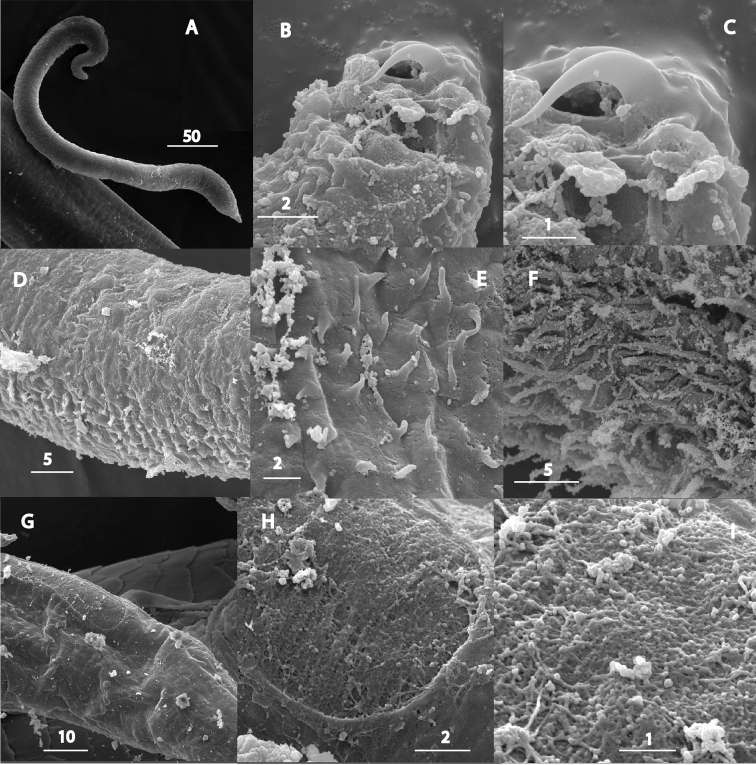
*Synoecnemahirsutum* Timm, 1959. SEM images. Male **A** entire worm **B-C** cephalic hooks **D-F** patches of cuticle covered with setae (**D-E** at mid-body **F** at anterior) **G-H** caudal organ at posterior **I** surface of caudal organ. Scales in µm.

Phylogenetic reconstructions were conducted from the alignment of 12 protein-coding genes with *Limuluspolyphemus* and *Lithobiusforficatus* as outgroups. For multiple alignments of AA sequences, the nucleotide sequences of each of the protein-coding genes were initially translated into AA with MEGA6. Conserved regions in the alignments of the 12 PCGs were selected using the GUIDANCE2 server (Sela et al. 2015). The final alignment included 3568 out of 3926 AA, representing 90.8% of the original sequence alignment. Optimal evolutionary models were chosen by PartitionFinder (Lanfear et al. 2012). Maximum Likelihood (ML) analysis was performed using IQ-TREE web server (Trifinopoulos et al. 2016) with 10,000 ultrafast bootstrap replicates (Hoang et al. 2018). Additionally, the coalescence-based analysis was carried out to test different phylogeny inference approaches. Phylogenetic analysis was performed for the datasets, based on separate protein-coding genes of the mitochondrial genome. The trees were visualised using TREEVIEW (Page 1996) and FIGTREE v.1.4.2 (http://tree.bio.ed.ac.uk/software/figtree/).

## ﻿Description

### ﻿ Synoecnema hirsutum

Taxon classificationAnimaliaSpiruridaUngellidae

Timm, 1959

6E79F360-FFCC-5097-952D-DE61BDDBEE46

#### General.

***Adults.*** Small nematodes lacking sexual dimorphism in body shape and of anterior end structure. Body almost cylindrical, tapering to both ends. Cuticle thin, transversely striated, bearing diverse short setae and spike-like outgrowths variously distributed in different specimens. Epidermis thick. Lateral fields absent. Anterior end curved bearing small paired cephalic hooks. Hooks sub-terminal, curved, thickened and directed dorsad. Hook base ca. 2 µm long, hook blades ca. 3 µm long, closely positioned with distal tips slightly diverging and directed towards or parallel to hook base. Cephalic sensilla indistinct. Amphids discernible in several specimens; apertures situated closely to hook base, elliptical, 1–2 µm wide. Mouth shifted ventrad; stoma absent. Pharynx clavate with long thin muscular corpus and large muscular-glandular pear-shaped terminal bulb displaced dorsally. Isthmus not expressed. Nerve ring encircling posterior of corpus. Excretory pore 1 µm wide, level with nerve ring, excretory duct strongly cuticularised, extending beyond bulb base, paired excretory canals weakly cuticularised, passing through very large excretory gland which can be tracked at least to mid-body. Intestine discernible at anterior, cardia-like structure present. Caudal organs long shallow grooves situated mid-laterally on the surface of posterior half of body; grooves’ surface lacking cuticle. No duct inside caudal organ observed. Tail extremity conical. Sexes often permanently in copula.

**Female**: N = 9. Body length = 1185 ± 138 (1014–1385) µm; a = 22.2 ± 3 (19–28); b = 11.2 ± 1.5 (9–13.2); max width = 64 ± 7 (55–75) µm; pharynx length = 107 ± 10 (93–123) µm; basal bulb height = 34 ± 3 (30–38) µm; basal bulb width = 19 ± 2 (16–22) µm; nerve ring from apex = 61 ± 10 (50–75) µm; excretory pore from apex = 74 ± 15 (54–101) µm; spermatheca from apex = 139 ± 18 (120–176) µm; V% = 44.5 ± 0 (36.1–49.5); egg length = 47 ± 2 (43–49) µm; egg width = 20 ± 1 (18–22) µm.

Anterior end tapering from pharyngeal base level. Pharyngeal procorpus 4–5 µm wide. Prodelphic, monodelphic. Postvulval body region very slightly swollen. Multilobed gland of obscure function present at posterior portion of body behind vulva, in some specimens hindering observation of gonad track and entwining gonad branches. Ovary distal cell situated close to tail extremity. Ovary running anteriad to the level of postvulval region, then turning posteriad to the point of distal cell and then turning again anteriad where it runs straight ahead until reflexing at some distance (about corresponding body diameter) behind pharynx base. At reflexion, gonad forming large, not distinctly offset spermatheca (av. size 56 µm × 34 µm), followed by thick-walled oviduct and spacious thin-walled uterus. Spermatheca filled with large spermatozoa ca. 2 µm in diameter. Vulva pre-equatorial, on slight protuberance, anterior vulval flap enlarged, vagina absent and vulva opens immediately into uterus. No post-uterine sack present. Eggs ovoid, arranged in a single row, 3–5 with fully-developed eggshells at a time. Fully-developed eggshells 1 µm thick densely covered with spikes 2 µm long. Tail pointing posterior to ovary distal cell; portion of tail free of gonad short. Rectum and anus indiscernible. Caudal organs extending from vulva level to nearly end of tail.

**Male**: N = 5. Length = 624 ± 77 (517–725) µm; a = 18.5 ± 0.7 (17.8–19.1); b = 5.3 ± 1.1 (3.9–6.1); c = 2.8 ± 9.5 (2.2–3.1); c’ = 7.9 ± 2.7 (6–10.9); max width = 36 ± 3 (32–38) µm; pharynx length = 129 ± 25 (103–167) µm; basal bulb height = 36 ± 3 (32–40) µm; basal bulb width = 18 ± 2 (15–20) µm; nerve ring from apex = 73 ± 1 (72–74) µm; excretory pore from apex = 73 ± 11 (60–82) µm; testis reflexion from apex = 316 ± 102 (260–497) µm; testis reflexion length = 64 ± 18 (40–83) µm; tail length = 240 ± 51 (201–328) µm.

Very similar to females in general appearance and morphology of anterior end and caudal organs, but much smaller and slimmer, especially at posterior. Monorchic. Testis reflexed at anterior third of body level. Flexure short and wide. Spermatocytes rounded, ca. 5 µm in diameter, arranged distally in two rows. Proximal part of reproductive system not distinctly differentiated into *vas deferens* and ejaculatory duct. Spicular apparatus and gubernaculum absent. Anal flaps developed unequally, anterior flap inflated and hook-like, while posterior one much smaller, partly overhanging indentation posterior to anus. No caudal sensilla detected. Caudal organ structure and position similar to that of females.

##### ﻿Remarks on morphology

The examination of the present species has shown its strong similarity to *S.hirsutum* Timm, 1959 in the general morphology, i.e. body proportions, the shape and size of cephalic hooks and eggs and the cuticle appearance. In terms of morphometrics, there are a few smaller differences which include: the slightly larger body size (1014–1385 µm vs. 0.70–1.09 mm, females and 517–725 µm vs. 512–654, males), the longer male tail (201–328 µm vs. 160–244 µm) and the slightly more posterior vulva position (36.1–49.5 vs. 34.8–46.5%) (see Table 1). Contrary to Timm (1959), we did not observe an anus in any female specimen. Therefore, we concluded it was absent or indistinct. Describing *S.anseriforme* and *S.hirsutum*, Timm (1959) discovered in females a median ventral pore located posterior to a vulva. The pore was interpreted by the author as an additional excretory pore due to the absence of a distinct intestine and rectum in these nematodes. Later, Timm (1966) re-appraises such a ventral pore of *S.anseriforme* as an anal aperture, but does not mention *S.hirsutum*. He also notes that an anal aperture is absent in the rest of the *Synoecnema*.

For *S.anseriforme* females, Timm (1959) also describes pocket-like phasmids or “suckers” and depicts similar structures for a female of *S.hirsutum*, but after re-examination of all known *Synoecnema* species of Baylis (1934) and Timm (1959, 1962), comes to the conclusion that “all have small cephalic hooks and lack distinct suckers in both sexes, with the exception of *S.drawidae* Baylis, 1934 in which the female has long narrow suckers with a slit-like opening”. In many illustrations to the species of *Synoecnema*, both of Baylis and Timm, the long narrow slits were depicted (though not explained) extending along the posterior body portion of a nematode. Further on, Ivanova and Spiridonov (1987), Ivanova and Pham Van Luc (1989) and Spiridonov (1993) described similar structures in *Synoecnema* species using light microscopy as long, slit-like caudal organs surrounded by a modified (fibrous) tissue. Structure of similar slits/grooves in *S.watinagii* and an undescribed *Synoecnema* from pheretimoid hosts was examined with the aid of scanning microscopy (Ivanova et al. 2017; unpubl.). The slits were deep (immersed in body wall) or shallow, all located mid-laterally and extended from mid-body to near end of the tail. The surface of a slit was devoid of a cuticle. No sucker-like structures were observed.

**Figure 3.**
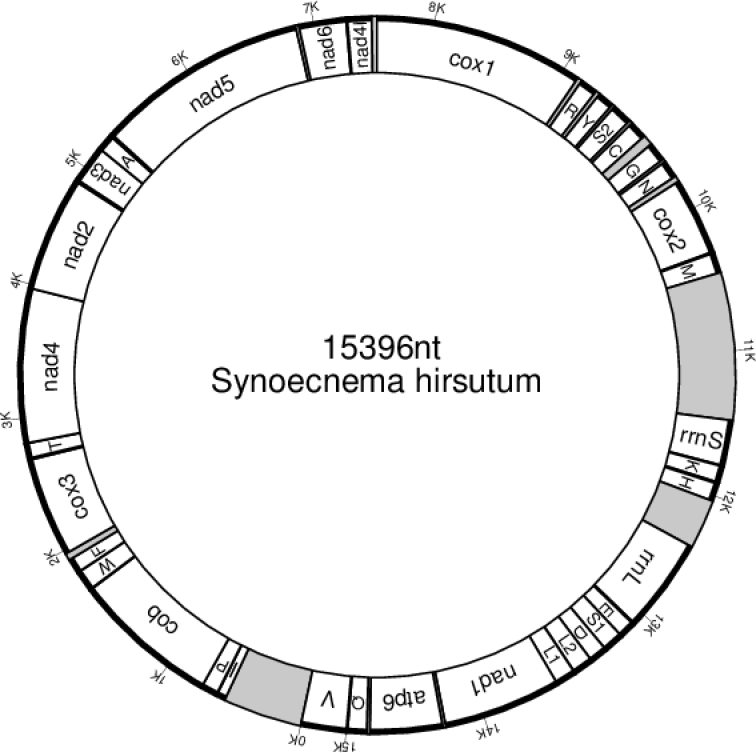
Map of the mitochondrial genome of *Synoecnemahirsutum*. Non-coding areas are shaded.

As the chief diagnostic feature of *S.hirsutum* seems to be the presence of setae covering the body of the nematode which also is a characteristic trait of the *Synoecnema* from our material, we tend to assume that both nematodes belong to the same species.

The term “caudal organ” was used when describing caudal structures of yet unknown function in nematodes of Drilonematoidea (Spiridonov et al. 2007; Ivanova and Neuhaus 2009; Ivanova and Bain 2013). Ivanova et al. (2017) discussed its possible function and pointed out the uniqueness of this structure in *Synoecnema* along with the simplifying of many other morphological traits.

##### ﻿Multigene analysis of *Synoecnemahirsutum* phylogenetic relationships

**Figure 4.**
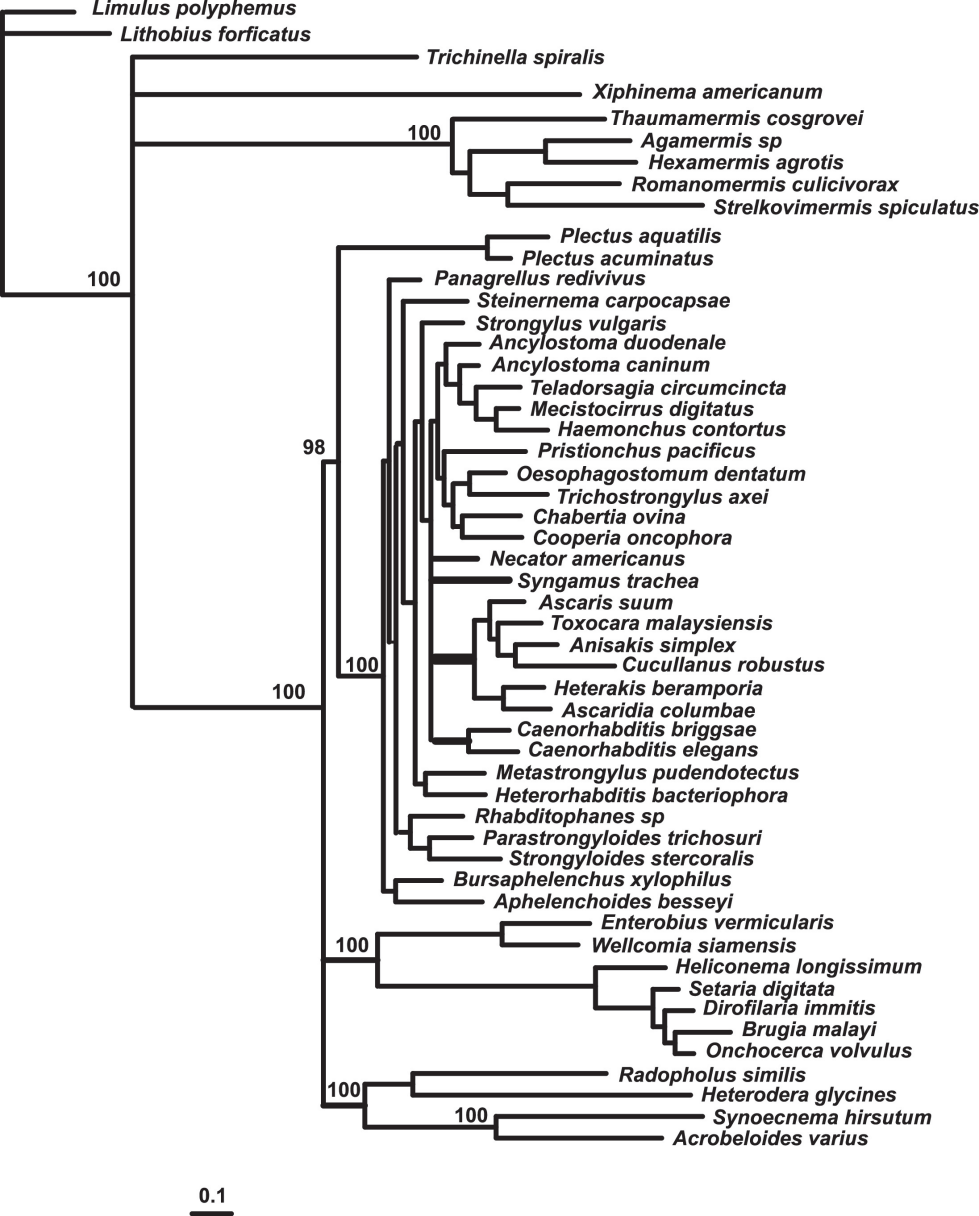
Phylogenetic relationships of *Synoecnemahirsutum* inferred from Maximum Likelihood analysis of concatenated dataset of 12 mitochondrial protein-coding genes.

The results of the multigene phylogenetic analysis of *Synoecnemahirsutum* relationships are presented in Fig. 4. The monophyly of Chromadoria nematodes, included in this analysis, is strongly supported as also the monophyly of the phylum Nematoda (Fig. 4). In this ML phylogenetic tree, *S.hirsutum* formed a clade under maximal bootstrap support with a soil free-living nematode *Acrobeloidesvarius* (Kim et al. 2020). As the mitochondrial data for the only other acrobelid studied, *A.complexus* (KM192361), are incomplete, they were not used in the analysis. The clade *Synoecnema*+*Acrobeloides* clustered with tylenchids under maximal bootstrap support. Contrary to expectations, the combined clade of tylenchids + *Synoecnema + Acrobeloides* remained outside of the larger clade containing all other Rhabditida (sensu De Ley and Blaxter 2002) and the two studied Plectidae (*Plectusaquatilis* and *P.acuminatus*). Additionally, a clade containing oxyurids and spirurids was in a similarly detached position. Such topology with the basal position of the clade (tylenchids + *Synoecnema + Acrobeloides*) was also observed in the trees inferred from the analysis of single mitochondrial genes *atp*6, *cox*I, *co*b, *nad*3, *nad*5 and *nad*6 (data not shown). The phylograms inferred from analyses of other mitochondrial genes (*cox*2, *cox*3, *nad*1 *nad*2, *nad*4 and *nad*4*L*) demonstrated more traditional basal position of plectids vs. all other representatives of Rhabditida. Coalescent analysis (not shown) demonstrated the great similarity with the ML tree (Fig. 4).

##### ﻿An arrangement of the genes in the mitochondrial genome of *Synoecnemahirsutum*

The circular molecule of complete mitochondrial genome has been reconstructed from separate contigs (Fig. 3). With the length of 15396 bp, this circular genome contains 12 protein-encoding genes, 22 transport RNA genes, two ribosomal sequences and three non-coding regions.

## ﻿Discussion

The superfamily Drilonematoidea is a taxon representing a quite exotic and not easily obtainable group of parasites. Due to little or no molecular data available for the majority of their taxa, relationships within four families constituting Drilonematoidea are still not phylogenetically resolved. Likewise, it is true concerning two subfamilies of Ungellidae (Ungellinae and Synoecneminae) where the present species belongs. The family Ungellidae has been split into Synoecneminae accommodating species lacking male spicular apparatus and Ungellinae housing ones with spicules and a gubernaculum (Spiridonov and Ivanova 2005). While no molecular data are available for Ungellinae, a limited number of molecular data was obtained for three out of 10 genera of Synoecneminae, namely *Synoecnema*, *Siconema* and *Drasico* Ivanova, Ganin & Spiridonov, 2014. The phylogenetic analysis, based on the D2-D3 region, confirmed the validity of all three genera (Ivanova et al. 2014, 2015).

Limited molecular data are available for some of the other higher taxa of Drilonematoidea and include several SSU and LSU sequences for two representatives of Homungellidae and representatives of three genera of Drilonematidae. The previous phylogenetic analyses were not in full agreement with the classification, based on morphology and topologies, showing the different positions on the phylogenetic tree for parasites from lumbricid and non-lumbricid earthworm hosts. The ‘lumbricid’ parasites were presented by *Dicelis* species from the Drilonematidae family (*D.lovatiana* Ivanova, 1993; *D.kimmeriensis* Ivanova, 1993; *D.rubidi* Ivanova, 1994; *D.caledoniensis* Spiridonov, Ivanova & Wilson, 2005; *D.ussuriensis* Spiridonov, Ivanova & Wilson, 2005) and were separated from the rest of Drilonematoidea phylogenetically representing a terminal branch of cephalobid phylogeny (Spiridonov et al. 2005; Ivanova and Spiridonov 2016). The rest of the sequences presented by ‘non-lumbricid’ parasites grouped together, uniting different families of Drilonematoidea.

In the present study, we aimed to overcome the limitations of the previous molecular phylogenetic analyses using a multigene analysis, based on 12 protein-encoding genes. Earlier, it has been shown that some mitochondrial genes are not suitable for the analysis of phyletic links between higher taxa because of saturation (Blouin et al. 1998). Still, we expected the wider set of genes to bring sufficient discriminatory power to reveal the phylogenetic relationships within Drilonematoidea. The close position of the ungellid *Synoecnemahirsutum* to the cephalobid *Acrobeloidesvarius* in the present analysis corresponds to the previous topologies (Ivanova et al. 2014, 2015), based on 18S and 28S rDNA. Some details of the phylogenetic tree contradict the traditional views on the nematode phylogeny. Thus, in the multigene phylogram (Fig. 4), plectids were found clustering only with a certain part of the order Rhabditida (precisely with the representatives of infraorders Ascaridomorpha, Ungellinae, Panagrolaimomorpha, Ungellinae and aphelenchids). Oxyuridomorpha, Spiruromorpha and tylenchids, along with the cephalobid *Acrobeloidesvarius* and the ungellid *Synoecnemahirsutum*, were closer to the base of the nematode tree, thus making the order Rhabditida (sensu De Ley and Blaxter 2002) polyphyletic. Such topology was supported in some phylograms inferred from single mitochondrial genes (*atp*6, *cox*I, *co*b, *nad*3, *nad*5 and *nad*6), but not supported in other (*cox*2, *cox*3, *nad*1 *nad*2, *nad*4 and *nad*4*L*). The multigene tree also demonstrated several patterns not common for traditional (pre-molecular) taxonomy of nematodes. In this tree, Tylenchoidea and Aphelenchoidea formed independent, not closely related clades, thus showing the polyphyly of Tylenchina. Such a phylogenetic pattern was reported in other analyses of nematode phylogeny, based on complete mitochondrial genomes (Sun et al. 2014; Kim et al. 2015). It can be added that, in the phylogenies inferred from the analysis of 18S rDNA sequences (van Megen 2009), the tylenchid nematodes and the majority of aphelenchids formed two independent clades (clade 10B vs. 12A+12B). The previously-reported polyphyly of Spirurina (Liu et al. 2013) is even less surprising, as very few (if any) morphological features speak in favour of the entity of this morphologically- and ecologically-diverse group. In our phylogram, previously reported splitting of true spirurids vs. the clade (ascaridids + cucullanids + heterakids) was also evident.

**Figure 5.**
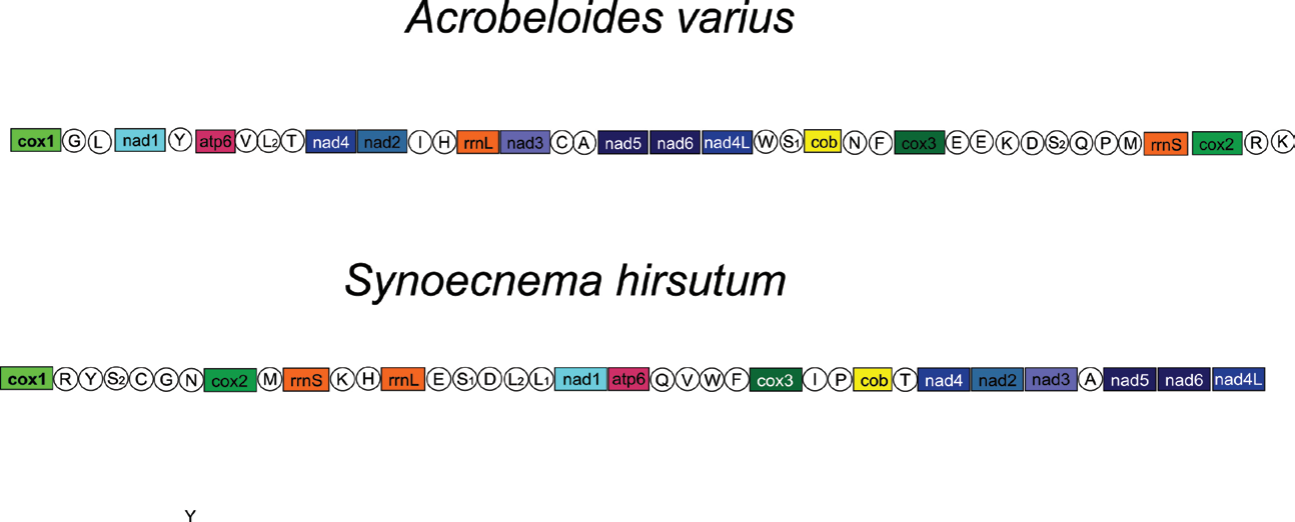
Linear maps of the mitochondrial genomes of *Synoecnemahirsutum* and *Acrobeloidesvarius*.

Previous attempts to find the place for ungellids and other Drilonematoidea in the system of Nematoda were based on nuclear ribosomal sequences only (Spiridonov et al. 2005, 2007; Ivanova and Spiridonov 2011; Ivanova et al. 2012; 2014;). First, LSU and SSU analyses had confirmed that the earthworm parasites from the family Creagrocercidae were not related to the rest of Drilonematoidea (Drilonematomorpha of De Ley and Blaxter 2002), but appeared to be close to *Domorganus* Goodey, 1947 (Ohridiidae, Plectida) (Ivanova, Spiridonov 2011). Further on, it was shown that at least two evolutionary lines of Drilonematoidea were related to Cephalobomorpha. Each of the lines, according to these phylogenetic reconstructions, evidently has independent roots in the free-living representatives of this infra-order. The obtained results, inferred from the multigene analysis of *S.hirsutum* in this study, support the previous topology, showing its close relations to the cephalobid *A.varius*. The clade, consisting of these two nematodes, was strongly supported (100%) in the ML tree. The length of branches leading to these nematodes and related tylenchids is quite long, which can be regarded as a sign of lengthy independent evolution. As biological traits of *S.hirsutum* and *A.varius* are markedly different, it is reasonable to question whether they can be the result of so-called a ‘long branch attraction’. Still, we believe that the obtained topology reflects the true phylogenetic relationships between ungellids and cephalobids. Common features in the arrangement of genes in the mitochondrial genome can be considered as an additional argument in this case. Indeed, only *A.varius* and *S.hirsutum* have a joint cluster of genes *nad*5 - *nad*6 - *nad*4L (Fig. 5). Though joint genes *nad*6 and *nad*4L are characteristic for different Ungellinae, Ascaridomorpha and Ungellinae (Kim et al. 2020), the junction of three genes without intermittent transport RNA genes makes it a unique feature of these ungellids and cephalobids.

## ﻿Data availability

The assembled mitochondrial genome is deposited in NCBI GenBank under accession MG294556. Protocols are deposited in protocols.io under: https://doi.org/10.17504/protocols.io.bv4gn8tw

## Supplementary Material

XML Treatment for Synoecnema hirsutum
